# Neural representation of proactive and reactive inhibitory control adaptations across sensory modalities

**DOI:** 10.1093/cercor/bhaf272

**Published:** 2025-10-30

**Authors:** Fumiko Nakajima, Koyuki Ikarashi, Koya Yamashiro, Genta Ochi, Tomomi Fujimoto, Daisuke Sato

**Affiliations:** Sports Physiology Laboratory, Department of Health and Sports, Faculty of Health Science, Niigata University of Health and Welfare, 1398 Shimami-cho, Kita ward, Niigata, Niigata, 950-3198, Japan; Major in Health and Welfare, Graduate School of Niigata University of Health and Welfare, 1398 Shimami-cho, Kita ward, Niigata, Niigata, 950-3198, Japan; Sports Physiology Laboratory, Department of Health and Sports, Faculty of Health Science, Niigata University of Health and Welfare, 1398 Shimami-cho, Kita ward, Niigata, Niigata, 950-3198, Japan; Institute for Human Movement and Medical Sciences, Niigata University of Health and Welfare, 1398 Shimami-cho, Kita ward, Niigata, Niigata, 950-3198, Japan; Sports Physiology Laboratory, Department of Health and Sports, Faculty of Health Science, Niigata University of Health and Welfare, 1398 Shimami-cho, Kita ward, Niigata, Niigata, 950-3198, Japan; Institute for Human Movement and Medical Sciences, Niigata University of Health and Welfare, 1398 Shimami-cho, Kita ward, Niigata, Niigata, 950-3198, Japan; Sports Physiology Laboratory, Department of Health and Sports, Faculty of Health Science, Niigata University of Health and Welfare, 1398 Shimami-cho, Kita ward, Niigata, Niigata, 950-3198, Japan; Institute for Human Movement and Medical Sciences, Niigata University of Health and Welfare, 1398 Shimami-cho, Kita ward, Niigata, Niigata, 950-3198, Japan; Sports Physiology Laboratory, Department of Health and Sports, Faculty of Health Science, Niigata University of Health and Welfare, 1398 Shimami-cho, Kita ward, Niigata, Niigata, 950-3198, Japan; Institute for Human Movement and Medical Sciences, Niigata University of Health and Welfare, 1398 Shimami-cho, Kita ward, Niigata, Niigata, 950-3198, Japan; Institute of Health and Sport Sciences, University of Tsukuba, 1-1-1 Tennodai, Tsukuba, Ibaraki 305-8574, Japan; Advanced Research Initiative for Human High Performance (ARIHHP), University of Tsukuba, 1-1-1 Tennodai, Tsukuba, Ibaraki 305-8574, Japan

**Keywords:** behavioral adaptation, neural adaptation, proactive slowing and reactive inhibition, athletes, sensory modality

## Abstract

Inhibitory control takes multiple forms, including proactive slowing, a strategic delay of responses, and reactive inhibition, the cancelation of an initiated response. How these processes adapt across sensory modalities and their neural mechanisms remains unclear. This cross-sectional study tested whether proactive slowing and reactive inhibition are adaptable across sensory modalities and supported by shared and distinct neural adaptations. We recruited 23 athletes and 21 age-matched controls. Participants performed a choice-reaction task, requiring rapid response selection, and a stop-signal task, requiring occasional cancelation of initiated responses. Proactive slowing and reactive inhibition were assessed across visual, auditory, and somatosensory modalities. Proactive slowing was measured by anticipatory response slowing, and reactive inhibition by stop-signal reaction time. Event-related potentials (ERPs) were recorded to examine neural processing. Athletes exhibited greater proactive slowing and reactive inhibition than controls across all modalities. ERP analyses revealed that proactive slowing was associated with greater N2 and smaller P3 amplitudes in athletes, suggesting enhanced early conflict monitoring and reduced reliance on later attentional control. Athletes showed greater N2 amplitudes for reactive inhibition, indicating superior stimulus-driven inhibition, similar to proactive slowing. These findings provide novel evidence that both proactive slowing and reactive inhibition adapt across sensory modalities, accompanied by neural changes that are partly shared (N2) and partly distinct (P3).

## Introduction

Behavioral adaptation is the ability to flexibly control actions proactively in anticipation of a task or reactively in response to sudden environmental changes. A critical component of behavioral adaptation is inhibitory control, which consists of interference control, proactive inhibition, and reactive inhibition (Bari and Robbins 2013; Diamond 2013; Kenemans 2015; van Rooij et al. 2024). Proactive inhibition refers to the potentiation of an inhibitory connection to the motor system in anticipation of a possible stop signal, such that any stop signal, when it occurs, has an enhanced impact. In line with recent accounts, proactive slowing can be considered the behavioral manifestation of proactive inhibition—the cognitive preparation to bias future stopping success (van den Wildenberg et al. 2022). In this sense, proactive-slowing reflects an internally regulated adjustment of response execution, distinct from a conscious waiting strategy in which participants deliberately withhold their responses until they are confident that no stop signal will occur. Although both are anticipatory, proactive inhibition engages neurocognitive mechanisms of control, while proactive slowing is a more observable behavioral manifestation of caution. Conversely, reactive inhibition is a stimulus-driven, bottom-up process that is engaged once a stop signal is presented, leading to the abrupt cancelation of an ongoing response (Braver 2012). These processes dynamically interact through cortico-basal ganglia circuits to facilitate flexible and efficient control of action. Their balance and effectiveness can be shaped by prior experiences, training, or neurological conditions, reflecting plastic changes in underlying neural representations (Aron 2011; Stuphorn and Emeric 2012; Alatorre-Cruz et al. 2021). Although converging evidence supports the existence of shared and distinct mechanisms across these inhibitory processes (Hester et al. 2004; Stuphorn and Emeric 2012), how each inhibitory process adapts remains poorly understood. As the relevance of these inhibitory control processes has been debated (Chikazoe et al. 2009; Zandbelt and Vink 2010), understanding the adaptation process in greater depth could provide valuable insights and highlight the significance of inhibitory control in behavioral adaptation.

Previous studies have also indicated that these inhibitory processes (PI, proactive slowing, and reactive inhibition) are influenced by sensory modalities (Falkenstein et al. 2002; Nieuwenhuis et al. 2004). For instance, auditory and somatosensory inputs have been shown to significantly influence PI and proactive slowing (Ikarashi, Sato, Fujimoto, et al. 2022a; Ikarashi, Sato, Ochi, et al. 2022b). Conversely, findings on reactive inhibition have been inconsistent, with some studies suggesting that auditory inputs are the most influential inhibitory cues (Ramautar et al. 2006; Carrillo-de-la-Peña et al. 2019), whereas others report no significant differences among visual, auditory, and somatosensory inputs (Ikarashi, Sato, Fujimoto, et al. 2022a). These findings suggest that modality-specific or supra-modal neural adaptations may underlie these inhibitory processes.

Cognitive tasks performed during electrocortical recordings have been used to investigate the neurocognitive mechanisms underlying task performance and to provide potentially relevant theoretical insights into these inhibitory processes. The event-related potential (ERP) technique, owing to its high temporal resolution, is widely used to examine the time course of adaptive inhibitory processes (Kok et al. 2004; Huster et al. 2010). Two major ERP components associated with inhibitory processes are the N2 negative peak at the frontocentral site, occurring approximately 200 to 300 ms after stimulus onset, and the P3 positive peak at the frontal and centroparietal sites, occurring approximately 300 to 500 ms post-stimulus (Enriquez-Geppert et al. 2010; Huster et al. 2020). The N2 component is believed to reflect conflict monitoring, error processing (Ramautar et al. 2006; Senderecka et al. 2018), and attentional orienting to salient or unpredicted stimuli (González-Villar et al. 2022), whereas the P3 component is associated with response inhibition (Tatz et al. 2021), conflict during ongoing motor execution (González-Villar et al. 2022), and attentional resource allocation (Ikarashi, Sato, Fujimoto, et al. 2022a). However, the relationship between the P3 component and inhibitory control remains controversial, as several studies have suggested that the P3 may reflect earlier stages of sensory processing (Huster et al. 2020) and may occur after the onset of muscle markers of inhibition (Jana et al. 2020). These interpretations are consistent with prior theoretical and empirical accounts (Kenemans 2015; Verbruggen et al. 2019; van Rooij et al. 2024), although some aspects remain debated. Consequently, the current evidence is insufficient to elucidate the role of P3 adaptation in inhibitory processes fully. Therefore, evaluating both ERP components (N2 and P3) may provide valuable insights into the neural representations underlying adaptation in each inhibitory process. In this study, we examined both the amplitudes and latencies of these ERP components, as they provide complementary insights into the underlying neurocognitive mechanisms: amplitudes reflect the magnitude of neural resource allocation to functions such as conflict monitoring and attentional control, whereas latencies index the temporal dynamics and processing speed of these operations.

Athletes have been reported to exhibit superior inhibitory control (Albaladejo-García et al. 2023; Fleddermann et al. 2023), particularly when driven by visual information (Chen et al. 2019; Xu et al. 2022), likely due to extensive long-term training and the repeated engagement of inhibitory control during competitive performance. This enhancement is thought to be associated with reduced N2 and P3 amplitudes, reflecting a lower neural cost for conflict monitoring, attentional orienting, and response inhibition (Kok et al. 2004; Ramautar et al. 2006; González-Villar et al. 2022). Such adaptations have also been observed in athletes engaged in interpersonal sports such as fencing, tennis, and handball, where rapid reactions and the frequent inhibition of pre-initiated responses are crucial (Zhang et al. 2015; Brevers et al. 2018; Heppe and Zentgraf 2019; Bravi et al. 2022). However, most of these studies have focused on a single sensory modality and have not distinguished among inhibitory processes. To address these limitations, recent studies have proposed methods for assessing inhibitory processes across multiple sensory modalities (Ikarashi, Sato, Fujimoto, et al. 2022a). Based on previous studies, examining inhibitory processes across multiple modalities and their associated neural mechanisms in athletes may contribute to a better understanding of these adaptations.

Among the three inhibitory processes, proactive slowing and reactive inhibition were selected as the primary focuses of the present study. These processes reflect functionally distinct yet complementary forms of inhibitory control—strategic modulation of response timing and rapid, stimulus-driven response cancelation—and are supported by partially dissociable neural circuits. Targeting proactive slowing and reactive inhibition facilitates a more sophisticated examination of the neurocognitive mechanisms underlying adaptive inhibitory control across anticipatory and reactive domains. Therefore, this study aimed to investigate behavioral and neural adaptations in athletes engaged in interpersonal competitive sports by comparing two inhibitory processes triggered by multimodal sensory inputs, using choice reaction and stop-signal tasks (SSTs). We hypothesized that proactive slowing and reactive inhibition would exhibit adaptations across sensory modalities, supported by both shared and distinct neural mechanisms. Specifically, we conceptualized the N2 component as an index of conflict monitoring (Ramautar et al. 2006; Senderecka et al. 2018) and attentional orienting (Polich 2007; Corbetta et al. 2008), which may operate in a supra-modal manner and thus show consistent patterns across inhibitory processes and sensory modalities. In contrast, the P3 component was interpreted as reflecting inhibitory control and attentional resource allocation, which may vary depending on task demands and potentially show modality-specific patterns.

## Materials and methods

### Participants

All experiments performed in this study conformed to the principles of the Declaration of Helsinki and were approved by the Ethics Committee of Niigata University of Health and Welfare, Japan (approval number: 18777-211,126). Written informed consent was obtained from all participants. The sample size was calculated using Superpower (Lakens and Caldwell 2021), which indicated that 20 participants per group would provide 85% power to detect an effect size of f = 0.25. This effect size was determined a priori based on both empirical and theoretical considerations. Previous studies investigating inhibitory control in athletes reported effect sizes in the range of 0.20 to 0.30 (Brevers et al. 2018; Yamashiro et al. 2023). Based on these findings and the conventional classification by Cohen (2013), we adopted f = 0.25 as a representative medium effect size. This approach follows the recommendations of Lakens (2022) for sample size justification when precise prior estimates are unavailable. As athletes from interpersonal sports have been reported to have superior inhibitory control in previous studies (Taddei et al. 2012; Zhang et al. 2015), we recruited kendo athletes for this study. The exclusion criteria were as follows: participants with a history of neurological or psychiatric disorders; female participants using prescription medications or hormonal contraception; left-handed participants; and those with abnormal or uncorrected vision. A total of 25 kendo athletes (12 female) from the same university kendo team (athlete [ATH] group) and 22 age-matched healthy controls (nine female) without previous sports or music training (control [CON] group) participated in the study.

To control for potential confounding effects, participants with formal music training were excluded from both groups, as previous studies have reported that such training can enhance executive functions, including inhibitory control (Moreno et al. 2011; Zuk et al. 2014). Due to scheduling conflicts, two participants in the ATH group and one in the CON group withdrew from the study. Table 1 shows the participant characteristics. All experiments involving female participants were conducted during the follicular phase, immediately after menses and before the rise in estradiol levels. This timing was chosen to minimize hormonal variability and to avoid the preovulatory phase, during which inhibitory control has been shown to decrease and sex differences to emerge (Colzato et al. 2010). In addition, our previous study demonstrated no significant sex differences in inhibitory control during the follicular phase (Ikarashi, Sato, Fujimoto, et al. 2022a), further supporting the use of this window for testing. Female participants were instructed to record their basal body temperature every morning upon waking to track their menstrual cycle. Two menstrual cycles preceding the first experiment were monitored to allow accurate estimation of menstrual cycle length. No irregularities in menstrual cycles were observed during the 2 mo preceding the study. All measurements were conducted between 9 AM and 1 PM to account for circadian variations.

**Table 1:** Participant characteristics.

	ATH group	CON group
Number (n)	23	21
Female	11	9
Male	12	12
Age (years)	21.00 ± 3.90	20.95 ± 3.76
Height (cm)	165.61 ± 5.71	164.95 ± 10.68
Weight (kg)	60.93 ± 10.03	61.10 ± 14.37
BMI	22.16 ± 3.02	22.33 ± 4.34
History of Kendo (years)	13.39 ± 5.81	
Competition level (n)		
Highly trained/national	8	
Trained/developmental	15	

Note: Classification of competition levels was defined in accordance with previous studies (McKay et al. 2022). BMI, body mass index; ATH, athlete group; CON, control group.

### Procedure

**Fig. 1:**
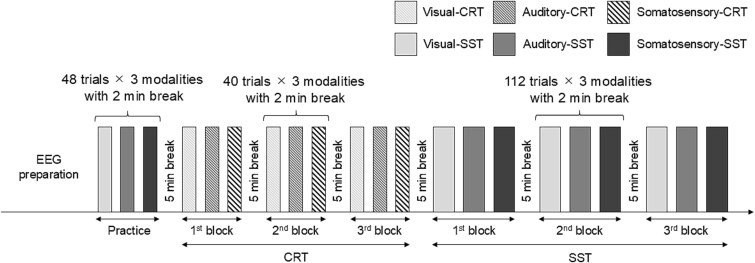
Experimental procedure in the present study. The participants first completed a practice block consisting of 48 trials of the SST in the visual, auditory, and somatosensory modalities, with a 2 min break. After a 5 min break, the participants then attempted three CRT blocks, which consisted of 40 trials for each modality, with a 2 min break. After a 5 min break, the participants then conducted three SST blocks, each consisting of 112 trials per modality, with a 2 min break. Among these, 84 (75%) were go trials, and 28 (25%) were stop trials. The order of the modalities was randomized across participants and counterbalanced between the two groups.

All participants were instructed not to engage in vigorous physical activity, consume alcohol, or smoke for 24 h before each experiment. Participants performed a choice reaction task (CRT) and a SST using visual, auditory, and somatosensory stimuli, referred to as the visual-, auditory-, and somato-CRT and SST, respectively. The order of the CRT and SST sessions was fixed for all participants, with the CRT administered first, followed by the SST. The experiment included 48 practice trials of each SST, with 2 min breaks, to ensure participants understood the task rules, similar to a previous study (Ikarashi, Sato, Fujimoto, et al. 2022a). Because the SST consisted of CRT and infrequent stop trials, practice trials were conducted before the CRT session to ensure that participants performed both tasks under the same conditions. However, this procedure could potentially affect CRT performance. To mitigate this risk, participants were informed in advance that no stop signals would appear during the CRT session. This procedure is illustrated in Fig.  1. The CRT and SST were performed using a custom-built program (Stop-Signal Task Programme, Medical Try System Co., Ltd, Tokyo, Japan), controlled by a computer (Ikarashi, Sato, Fujimoto, et al. 2022a). Visual stimuli (white and red arrows) were presented using a custom-built LED panel (MTS207642-01785, Medical Try System Co., Ltd, Tokyo, Japan) to prevent signal delay. Auditory and somatosensory stimuli were delivered using earphones (YE103J, Medical Try System Co., Ltd, Tokyo, Japan), ring electrodes for Go signals (Finger Electrode NM-451B, NIHON KODEN Co., Tokyo, Japan), and bar electrodes for Stop signals (019-401,400, GE Healthcare, IL, USA).

## Choice reaction task

During each task, participants were instructed to place their right and left index fingers on the response pad (RB Series Response Pad RB-844, Cedrus Corp., CA, USA). All tasks began with an attentional auditory cue of 500 Hz at 80 dB, presented for 500 ms. In the visual CRT, participants were instructed to press the corresponding response pad button with their right or left index finger as quickly and accurately as possible when a right- or left-pointing white LED arrow was presented for 500 ms. In the auditory CRT, participants were instructed to press the corresponding response pad button with their right or left index finger when a tone of 1000 Hz at 80 dB was presented for 500 ms to the right or left ear. In the somato CRT, participants were instructed to press the corresponding response pad button with their right or left index finger when electrical stimuli with a pulse width of 200 μs were applied to the same finger using a ring electrode. The somatosensory stimulus intensity was fixed at 2.5 times the participant’s sensory threshold, ensuring perceptual clarity without eliciting pain, discomfort, or involuntary muscle activation. This stimulation–response configuration was employed in our previous studies (Ikarashi, Sato, Fujimoto, et al. 2022a; Yamashiro et al. 2023), and in both those studies and the present one, no participants reported any interference with motor responses due to the stimulation.

## Stop-signal task

**Fig. 2:**
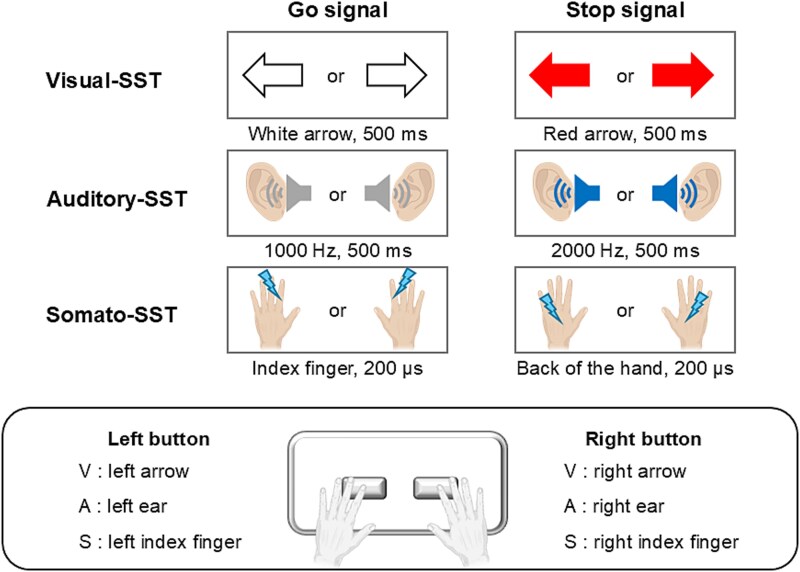
Stop-signal task paradigm in each modality. Similar to the CRT, participants were instructed to place their right and left index fingers on the response pad buttons during each task. For each Go trial, the Go signals followed the same protocol as SRT. Participants were instructed to press the button corresponding to the Go signals as quickly and accurately as possible using their right or left index fingers. In contrast, participants had to suspend their already initiated responses when a Stop signal was presented after an SSD during the Stop trials. In the visual-SST, the Stop signal was a red right- or left-pointing arrow presented after the SSD. In the auditory-SST, the Stop signal was a 2,000 Hz, 80 dB tone presented on the same side as the Go signal. In the somato-SST, the Stop signal, using electrical stimulation, was applied to the back of the hand on the same side as the Go signal.

Figure  2 illustrates the SST paradigm. Similar to the CRT, the Go and Stop trials in each task began with an auditory attentional cue (80 dB, 500 Hz) presented for 500 ms, followed by a 500 ms blank interval. Participants were instructed to place their right and left index fingers on the response pad. The Go signals in each task were the same as those in the CRT: visual—a right- or left-pointing white LED arrow presented for 500 ms; auditory—a 1,000 Hz tone at 80 dB delivered for 500 ms to the right or left ear; somatosensory—electrical stimuli with a pulse width of 200 μs applied to the right or left index finger. Participants were instructed to press the corresponding response pad button with their right or left index finger as quickly and accurately as possible when each Go signal was presented. The maximum response time was set to 1,500 ms (Devor et al. 2025). Participants were instructed not to wait for the presentation of a Stop signal on each trial and were reminded to respond quickly and accurately before progressing to the next block to minimize task noncompliance (Verbruggen et al. 2019). On 25% of trials, stop signals were presented after the stop-signal delay (SSD), and participants attempted to inhibit their response (Stop trial). The SSD, defined as the interval between the Go and Stop signals, was initially set at 250 ms and subsequently adjusted according to the participant’s performance. The variation was achieved using the staircase method, which aims to yield approximately 50% successful and 50% unsuccessful response-inhibited trials. If a Stop trial was successful or unsuccessful, the SSD in the subsequent Stop trial was increased or decreased by 50 ms, respectively (Logan et al. 1984). Hence, the difficulty of successful inhibition was adjusted accordingly: when the delay was short, the probability of inhibition was high, and vice versa. The entire duration of each trial was set to 3.0 s.

## Behavioral analysis

Proactive slowing and reactive inhibition were evaluated as previously described (Ikarashi, Sato, Fujimoto, et al. 2022a). Proactive slowing was evaluated using proactive slowing time (PST), calculated by subtracting the averaged reaction time in the CRT (C-RT) from that in the Go trials of the SST (Go-RT). A longer PST indicates greater proactive-slowing performance, reflecting delayed movement initiation in anticipation of a stop signal. Reactive inhibition was assessed using stop-signal RT (SSRT), an index of response inhibition efficiency. A shorter SSRT (Verbruggen et al. 2019) indicates less time required to inhibit a response and greater reactive-inhibition performance. The SSRT was determined using the integration method (Verbruggen et al. 2019) and calculated as follows: (i) Calculate *p*(response|signal), the probability of responding during a stop trial. (ii) Order the response times of all Go trials with a response, including omissions and unsuccessful trials (ie the RT distribution of Go trials). (iii) Calculate the nth RT as *n* = RT distribution × *p*(response|signal). (iv) SSRT = nth RT—mean SSD***.***

### Electroencephalogram recording and ERP analysis

A continuous electroencephalogram (EEG) was recorded from 10 electrodes (Fz, F3, F4, FCz, Cz, C3, C4, Pz, P3, P4) using the mastoids (M1–M2) as the reference, based on the 10 to 20 system during the CRT and SST sessions, as described in previous studies (Ikarashi, Sato, Fujimoto, et al. 2022a; Ikarashi, Sato, Ochi, et al. 2022b). EEG and electrooculograms (EOG) were recorded at a sampling rate of 2,500 Hz and filtered using a 0.1 to 100 Hz bandpass and a 50 Hz notch filter, with a Brain Products amplifier system and Brain Vision Professional Recorder (Brain Products GmbH, Germany). All electrode impedances were maintained below 5 kΩ. To remove artifacts caused by eye blinks, simultaneous EOG activity was recorded from the bilateral external canthi and the left infraorbital and supraorbital areas. EEG data were analyzed using Brain Vision Professional Analyzer 2 (Brain Products GmbH, Germany) after removing eye-blink and movement artifacts through visual inspection. Given the limited number of electrodes, independent component analysis (ICA)-based correction was not applied; instead, artifacts were removed manually through visual inspection to ensure the reliability of retained epochs, as recommended for low-density EEG recordings (Luck 2014).

In the offline analysis, a bandpass filter of 0.1 to 30 Hz was applied to the continuous EEG data, followed by downsampling to 500 Hz. The EEG data were epoched from 100 ms pre-stimulus to 500 ms post-stimulus—corresponding to the Go signal in the Go trials and the Stop signal in the SST trials—extracted separately and baseline-corrected using the pre-stimulus interval. Additionally, three trial types from the SSTs were identified and extracted from the EEG data: trials in which participants (i) successfully responded after a Go cue, (ii) successfully inhibited their response after a Stop cue (successful stop, SS), or (iii) failed to inhibit their response after a Stop cue (unsuccessful stop, US). Any epochs contaminated with artifacts exceeding amplitudes of ±100 μV were excluded from further analysis.

We extracted the signal-locked waveforms in the CRT and the Go- and Stop-signal-locked waveforms in the SST, termed CRT-, Go-, and Stop-ERP, respectively. Stop-signal-locked waveforms were further separated into SS and US trials (US- and SS-ERP). We calculated the waveform difference by subtracting the CRT from the Go-ERP (proactive-slowing ERP) to reveal neural processes related to proactive slowing. To examine the neural processes associated with differences in reactive inhibition, we subtracted the SS-ERP from the US-ERP (reactive-inhibition ERP; US minus SS). For these subtracted ERP waveforms, N2 latencies and amplitudes were measured at Fz, F3, F4, and FCz as the maximum negative values within the 100 to 300 ms time window. P3 latencies and amplitudes were measured at Fz, Cz, and Pz as the maximum positive values within the 200 to 500 ms time window (Carrillo-de-la-Peña et al. 2019; Ikarashi, Sato, Fujimoto, et al. 2022a).

## Statistical analysis

Statistical analyses were performed using the linear mixed-effects models (LMMs) function of the “lme4” package in R (version 4.2.2; R Core Team, Vienna, Austria) (RC Team 2013). The dependent variables were: (i) behavioral measures obtained from the CRT (C-RT), Go trials (Go-RT, % errors, and % misses), PST, and Stop trials (%SS, %US, SSD, and SSRT), and (ii) neurophysiological measures, including proactive-slowing and reactive-inhibition related ERP components (N2 and P3). The LMM was then fitted to the data using group, modality and electrode as fixed effects, and participant number (PNr) as a random effect, using the following equations for behavioral and neurophysiological data:

(i) dependent variable ~ (1|PNr) + group*modality
(ii) dependent variable ~ (1|PNr) + group*modality*electrode

For neurophysiological measures of N2 and P3 latencies and amplitudes—termed proactive-slowing N2 and reactive-inhibition N2, and proactive-slowing P3 and reactive-inhibition P3, respectively—we performed pairwise testing for each of our a priori hypotheses, following a previous report (Luck and Gaspelin 2017). In all models, both dependent and continuous independent variables were standardized. Spearman’s correlation analysis was performed to assess the relationship between proactive slowing and reactive inhibition. Additionally, Spearman’s correlation analysis was conducted to assess the relationships among sensory modalities in proactive slowing and reactive inhibition. To further examine the functional relevance of the ERP components showing group differences, neural and behavioral correlations were conducted to evaluate whether these neural indices are associated with individual differences in proactive slowing and reactive inhibition. *P*-values from these pairwise and correlation analyses were corrected for multiple comparisons using the Bonferroni correction. Statistical significance was set at *P* < 0.05.

## Results

### Behavioral performance across sensory modalities and groups

We first examined how response times and inhibitory control varied across sensory modalities and groups (ATH vs. CON).

For C-RT, the LMM revealed that C-RTs were shortest in the somatosensory modality, followed by the auditory (|b| = 13.832, SE = 1.876, t[484] = 7.371, *P* < 0.001), and then the visual modality (|b| = 16.448, SE = 1.876, t[484] = 8.765, *P* < 0.001) (Table 2). No significant difference was observed in C-RT between the ATH and CON groups.

**Table 2:** Behavioral data for each modality in ATH and CON groups.

	ATH group			CON group		
	Visual	Auditory	Somatosensory	Visual	Auditory	Somatosensory
CRT						
C-RT (ms)	236.40 ± 23.09	231.67 ± 34.13	221.12 ± 43.06	248.03 ± 30.64	247.74 ± 44.10	230.30 ± 38.80
SST						
Go trials						
% errors: commission	0.07 ± 0.26	0.19 ± 0.41	0.19 ± 0.52	0.43 ± 0.80	0.51 ± 0.55	0.25 ± 0.32
% misses: omission	0.24 ± 0.47	0.22 ± 0.60	0.67 ± 1.42	1.19 ± 2.11	0.49 ± 0.86	1.21 ± 2.03
Go-RT (ms)	454.06 ± 109.46	488.15 ± 103.79	526.62 ± 116.39	297.96 ± 41.13	341.11 ± 48.41	332.08 ± 62.97
PIT (ms)	217.66 ± 109.65	256.49 ± 97.47	300.86 ± 105.01	49.93 ± 24.18	93.38 ± 50.31	101.77 ± 55.45
Stop trials						
% SS	52.12 ± 5.23	54.20 ± 3.82	55.30 ± 4.81	44.56 ± 1.62	46.71 ± 2.55	46.54 ± 4.47
Mean SSD (ms)	282.38 ± 108.27	350.41 ± 86.23	374.92 ± 104.61	105.61 ± 42.92	186.31 ± 59.96	155.70 ± 75.18
SSRT	155.09 ± 26.52	114.96 ± 21.74	124.57 ± 23.13	185.74 ± 23.00	148.77 ± 29.96	165.16 ± 31.42

Notes: Mean ± SD; C-RT, choice reaction time; % Errors, the rate of wrong key press; % Misses, the rate of no response or reaction times exceeding a threshold.

Regarding the Go-RT in the SST, the ATH group showed significantly slower Go-RTs than the CON group, suggesting stronger proactive slowing (visual: |b| = 156.095, SE = 23.803, t[50.968] = 6.558, *P* < 0.001; auditory: |b| = 147.056, SE = 23.803, t[50.968] = 6.178, *P* < 0.001; somatosensory: |b| = 194.540, SE = 23.803, t[50.968] = 8.173, *P* < 0.001). Go-RTs also varied by modality, progressively decreasing in the order of visual, auditory, and somatosensory modalities (visual vs. auditory: |b| = 38.418, SE = 4.608, t[484] = 8.338, *P* < 0.001; visual vs. somatosensory: |b| = 54.22, SE = 4.608, t[484] = 11.768, *P* < 0.001; auditory vs. somatosensory: |b| = 15.802, SE = 4.608, t[484] = 3.43, *P* < 0.001).

Similar to Go-RTs, PSTs were longer in the ATH group (visual: |b| = 167.728, SE = 22.213, t[51.145] = 7.551, *P* < 0.001; auditory: |b| = 163.120, SE = 22.213, t[51.145] = 7.344, *P* < 0.001; somatosensory: |b| = 199.112, SE = 22.213, t[51.145] = 8.964, *P* < 0.001) and varied across modalities, with the longest PST in the somatosensory modality, followed by the auditory and visual modalities (visual vs. auditory: |b| = 41.03, SE = 4.327, t[484] = 9.482, *P* < 0.001; visual vs. somatosensory: |b| = 68.234, SE = 4.327, t[484] = 15.769, *P* < 0.001; auditory vs. somatosensory: |b| = 27.205, SE = 4.327, t[484] = 6.287, *P* < 0.001).

For SSRT, the ATH group exhibited significantly shorter SSRTs across modalities compared to the CON group (visual: |b| = 30.661, SE = 6.381, t[58.918] = 4.805, *P* = 0.001; auditory: |b| = 33.806, SE = 6.381, t[58.918] = 5.298, *P* < 0.001; somatosensory: |b| = 41.553, SE = 6.381, t[58.918] = 6.512, *P* < 0.001), indicating superior reactive inhibition, consistent with previous studies in the visual modality (Brevers et al. 2018; Wang et al. 2024). SSRTs were fastest in the auditory modality, followed by the somatosensory and visual (visual vs. auditory: |b| = 38.62, SE = 1.86, t[484] = 20.761, *P* < 0.001; visual vs. somatosensory: |b| = 26.284, SE = 1.86, t[484] = 14.13, *P* < 0.001; auditory vs. somatosensory: |b| = 12.336, SE = 1.86, t[484] = 6.632, *P* < 0.001), consistent with shorter SSRT in the auditory modality compared to the visual modality (Ramautar et al. 2006; Carrillo-de-la-Peña et al. 2019).

**Fig. 3:**
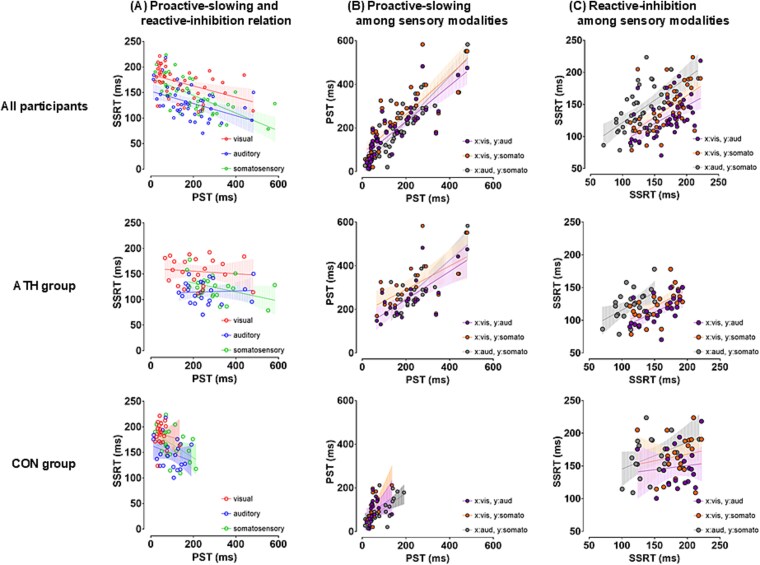
Relationships between proactive slowing and reactive inhibition and their cross-sensory modal associations. A) a negative correlation was observed between PST and SSRT across all participants, suggesting a trade-off between proactive-slowing and reactive-inhibition mechanisms. However, this relationship was abolished when analyzed within each group, potentially reflecting increased variability in inhibitory control strategies between individuals. Each dot represents an individual participant. B) Cross-sensory modality correlations of PST between the visual, auditory, and somatosensory modalities were examined in the ATH and CON groups. Significant positive correlations were observed in both groups, indicating that proactive slowing operates in a modality-general manner. C) Cross-sensory modality correlations of SSRT between the visual, auditory, and somatosensory modalities in the ATH and CON groups. Significant correlations were only found in the ATH group, suggesting that reactive inhibition may generalize across modalities as a result of long-term training. PST: Proactive-slowing time (Go-RT difference between SST and CRT); SSRT: Stop-signal reaction time; ATH: Athlete group; CON: Control group.

As shown in Fig.  3, PST and SSRT were negatively correlated across modalities in all participants, suggesting a trade-off between proactive slowing and reactive inhibition (visual: ρ = −0.475, *P* = 0.004; auditory: ρ = −0.483, *P* = 0.003; somatosensory: ρ = −0.705, *P* < 0.001), but not when analyzed separately for each group. Additionally, strong cross-sensory modality correlations were observed in PST across all participants (visual and auditory: ρ = 0.888, *P* < 0.001; visual and somatosensory: ρ = 0.873, *P* < 0.001; auditory and somatosensory: ρ = 0.889, *P* < 0.001), and were also evident in both the ATH (visual and auditory: ρ = 0.667, *P* = 0.002; visual and somatosensory: ρ = 0.513, *P* = 0.012; auditory and somatosensory: ρ = 0.657, *P* = 0.002) and CON groups (visual and auditory: ρ = 0.725, *P* < 0.001; visual and somatosensory: ρ = 0.668, *P* = 0.003; auditory and somatosensory: ρ = 0.703, *P* = 0.001). Moreover, cross-sensory modality correlations were shown in SSRT across all participants (visual and auditory: ρ = 0.544, *P* < 0.001; visual and somatosensory: ρ = 0.600, *P* < 0.001; auditory and somatosensory: ρ = 0.645, *P* < 0.001) and in the ATH group (visual and auditory: ρ = 0.617, *P* = 0.005; visual and somatosensory: ρ = 0.668, *P* = 0.001; auditory and somatosensory: ρ = 0.457, *P* = 0.028), but not in the CON group.

### ERP components related to proactive slowing and reactive inhibition

**Fig. 4:**
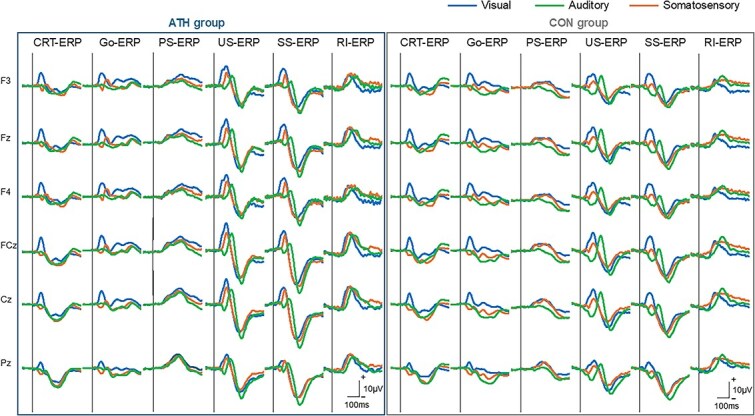
Grand-averaged ERPs and difference waveforms for proactive slowing and reactive inhibition grand-averaged ERPs for CRT, Go, US, and SS trials across the visual, auditory, and somatosensory modalities in the ATH and CON groups. ERPs are shown at six midline and fronto-central electrode sites (F3, Fz, F4, FCz, Cz, and Pz). Each column shows the stimulus-locked ERP for CRT trials (CRT-ERP), Go signal-locked ERP for SST trials (Go-ERP), stop signal-locked ERP for unsuccessful inhibition (US-ERP), and stop signal-locked ERP for successful inhibition (SS-ERP), presented separately for the ATH (left panel) and CON (right panel) groups. For clarity, the plotting polarity is explicitly indicated within the figure. The proactive-slowing related waveform is derived by subtracting CRT-ERP from Go-ERP, isolating neural activity related to proactive slowing (proactive-slowing N2 and P3). The reactive-inhibition related waveform is derived by subtracting SS-ERP from US-ERP (US minus SS), highlighting reactive-inhibition components (reactive-inhibition N2 and P3). These ERP waveforms provide visual confirmation of the time windows and scalp distributions used for subsequent statistical analyses (see Fig.  5).

**Fig. 5:**
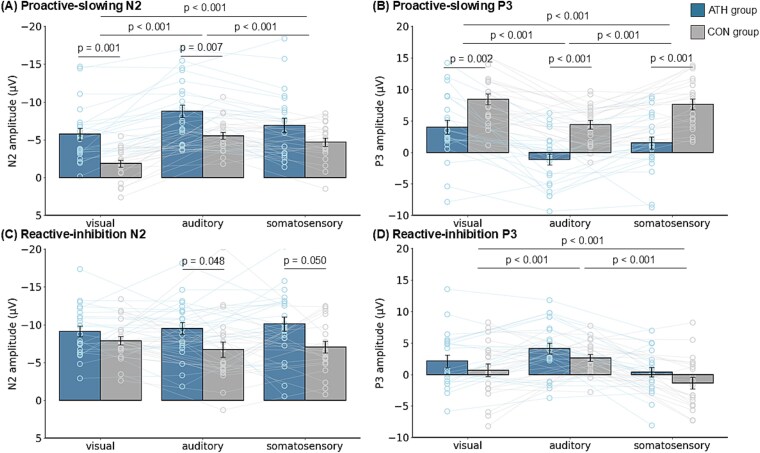
Amplitudes of proactive and reactive inhibition-related ERP in all sensory modalities. A) and B) show proactive-slowing N2 and P3 amplitudes, which indicate larger N2 and smaller P3 amplitudes in the ATH group than in the CON group and significant differences among sensory modalities. C) and D) present reactive-inhibition N2 and P3 amplitude, which indicate larger N2 amplitude in the ATH group than in the CON group, but no difference in P3 amplitude. In D), positive values denote US > SS. Because value magnitudes can be asymmetric across individuals, the sign frequency (counts of positive vs. negative values) may not fully align with the group’s central tendency; this may help explain the pattern observed in the auditory modality. All data are presented as raw values. Bidirectional whiskers indicate 95% confidence intervals.

We analyzed the N2 and P3 components to evaluate the neural activities related to proactive slowing and reactive inhibition. Figure  4 illustrates the grand-averaged ERP waveforms for CRT, Go, US, and SS trials, as well as the difference waveforms used to isolate components related to proactive slowing and reactive inhibition across the three sensory modalities (visual, auditory, and somatosensory). Waveforms are plotted for six representative electrode sites (F3, Fz, F4, FCz, Cz, and Pz) and for both groups (ATH and CON). This figure demonstrates modality-specific and group-specific differences in the N2 and P3 components that underlie proactive-slowing and reactive-inhibition processes. Notably, proactive-slowing ERP components (proactive-slowing N2 and P3) can be observed by subtracting CRT from Go trials, whereas reactive-inhibition related components (reactive-inhibition N2 and P3) are identified by subtracting SS from US trials. These waveforms help validate the component definitions used in the subsequent statistical analyses presented in Fig.  5. ERP plots adopt a positive-up polarity; accordingly, for the difference waves, positive proactive-slowing ERP values denote Go > CRT and positive reactive-inhibition ERP values denote US > SS.

#### Proactive slowing (proactive-slowing N2 and proactive-slowing P3)

Proactive-slowing N2 latency was significantly shorter in the CON group than in the ATH group, but only in the visual modality (|b| = 47.824, SE = 8.137, t[75.721] = 5.878, *P* < 0.001). No significant differences were found among the modalities in proactive-slowing N2 latency. Proactive-slowing N2 amplitude was larger in the ATH group in the visual (|b| = 3.977, SE = 0.923, t[53.341] = 4.308, *P* = 0.001) and auditory (|b| = 3.415, SE = 0.923, t[53.341] = 3.699, *P* = 0.007) modalities, but not in the somatosensory modality. Additionally, proactive-slowing N2 amplitude was smallest in the visual modality, followed by the somatosensory and auditory modalities (visual vs. auditory: |b| = 3.390, SE = 0.1996, t[484] = 16.982, *P* < 0.001; visual vs. somatosensory: |b| = 2.006, SE = 0.1996, t[484] = 10.048, *P* < 0.001; auditory vs. somatosensory: |b| = 1.384, SE = 0.1996, t[484] = 6.934, *P* < 0.001).

No group or modality effects were observed for proactive-slowing P3 latency. Proactive-slowing P3 amplitude was consistently smaller in the ATH group across all modalities (visual: |b| = 4.038, SE = 0.993, t[71.218] = 4.068, *P* = 0.002; auditory: |b| = 4.750, SE = 0.993, t[71.218] = 4.786, *P* < 0.001; somatosensory: |b| = 5.147, SE = 0.993, t[71.218] = 5.185, *P* < 0.001). Additionally, proactive-slowing P3 amplitude was smallest in the auditory modality, followed by the somatosensory and visual modalities (visual vs. auditory: |b| = 4.129, SE = 0.371, t[352] = 11.134, *P* < 0.001; visual vs. somatosensory: |b| = 1.507, SE = 0.371, t[352] = 4.065, *P* < 0.001; auditory vs. somatosensory: |b| = 2.621, SE = 0.371, t[352] = 7.069, *P* < 0.001).

#### Reactive inhibition (reactive-inhibition N2 and P3)

Reactive-inhibition N2 latency varied across modalities, with latencies progressively shorter in the order of visual, somatosensory, and auditory (visual vs. auditory: |b| = 50.034, SE = 3.006, t[484] = 16.643, *P* < 0.001; visual vs. somatosensory: |b| = 30.307, SE = 3.006, t[484] = 10.081, *P* < 0.001; auditory vs. somatosensory: |b| = 19.727, SE = 3.006, t[484] = 6.562, *P* < 0.001), but not between groups. Reactive-inhibition N2 amplitude was larger in the ATH group for the auditory (|b| = 3.202, SE = 1.048, t[66.632] = 3.055, *P* = 0.048) and somatosensory (|b| = 3.191, SE = 1.048, t[66.632] = 3.044, *P* = 0.050) modalities, but not in the visual modality. No significant differences were found across sensory modalities. Reactive-inhibition P3 amplitude was largest in the auditory modality, followed by the visual and somatosensory modalities (visual vs. auditory: |b| = 2.053, SE = 0.334, t[352] = 6.143, *P* < 0.001; visual vs. somatosensory: |b| = 1.454, SE = 0.334, t[352] = 4.349, *P* < 0.001; auditory vs. somatosensory: |b| = 3.507, SE = 0.334, t[352] = 10.492, *P* < 0.001), although this effect did not vary by group. No significant differences in reactive-inhibition P3 latency were found.

### Relationship between behavior and ERP components

We conducted correlation analyses to explore associations between behavioral indices (PST, SSRT) and ERP measures (N2, P3).

#### Proactive slowing

**Fig. 6:**
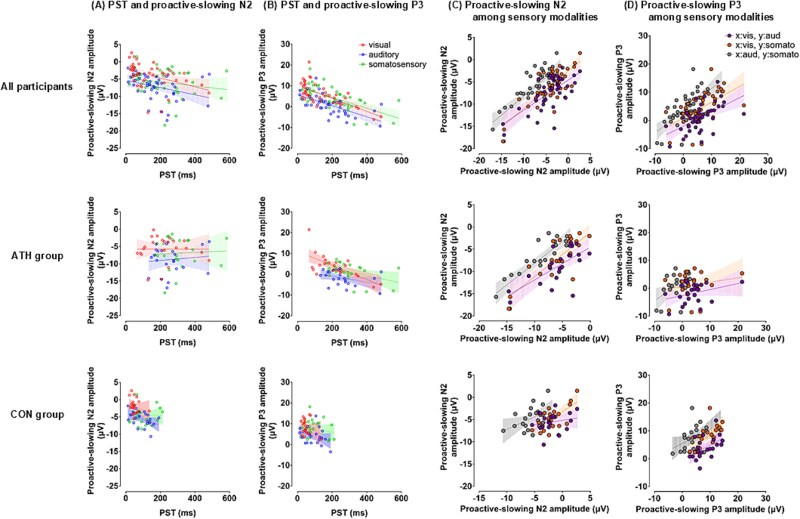
Associations between ERP components and behavioral indices, and cross-modal relationships of ERP amplitudes. A) Proactive-slowing N2 amplitudes were negatively correlated with PST in the visual and auditory modalities across all participants. However, this relationship was not consistently observed when analyzed separately for each group, except in the auditory modality of the CON group. B) Proactive-slowing P3 amplitudes showed significant negative correlations with PST across all sensory modalities in all participants, and similar associations were found in the ATH group. These results suggest that reduced attentional allocation (reflected by smaller P3 amplitudes) may contribute to greater proactive slowing. C) Proactive-slowing N2 amplitudes were positively correlated across sensory modalities in all participants and in the ATH group. This finding indicates that neural mechanisms related to conflict monitoring (as indexed by proactive-slowing N2) may support the adaptation of proactive slowing in a modality-independent manner, particularly in athletes. D) Proactive-slowing P3 amplitudes were positively correlated across sensory modalities in all participants and in the CON group. This suggests that individuals in the CON group may rely on shared attentional resources for proactive slowing, regardless of the sensory modality involved. Each dot represents an individual participant. Red, blue, and green circles and lines represent the visual, auditory, and somatosensory modalities, respectively.

Negative correlations were observed between PST and proactive-slowing N2 amplitude in the visual (ρ = −0.500, *P* = 0.006) and auditory (ρ = −0.431, *P* = 0.024) modalities. However, these relationships were not evident in the separate group analyses, except for the auditory modality in the CON group (ρ = −0.442, *P* = 0.045), as shown in Fig.  6A. PST was significantly negatively correlated with proactive-slowing P3 amplitude across all modalities in all participants (visual: ρ = −0.671, *P* < 0.001; auditory: ρ = −0.703, *P* < 0.001; somatosensory: ρ = −0.631, *P* < 0.001), and similar relationships were observed only in the ATH group (visual: ρ = −0.615, *P* = 0.002; auditory: ρ = −0.430, *P* = 0.041; somatosensory: ρ = −0.396, *P* = 0.061) as presented in Fig.  6B.

#### Reactive inhibition

Reactive-inhibition N2 amplitude showed positive correlations with SSRT in the auditory (ρ = 0.452, *P* = 0.016) and somatosensory (ρ = 0.503, *P* < 0.001) modalities; however, these relationships were not observed in the separate group analyses. Reactive-inhibition N2 latency was positively correlated with SSRT only in the visual modality (ρ = 0.540, *P* < 0.001); however, this correlation was not observed in the separate group analyses. No consistent correlations were observed for reactive-inhibition P3.

#### Cross-inhibitory process and cross-sensory modal relationships

N2 and P3 amplitudes were significantly correlated across both inhibitory processes, although the correlations were weak, as indicated by low correlation coefficients (proactive-slowing and reactive-inhibition N2: ρ = 0.222, *P* = 0.010; proactive-slowing and reactive-inhibition P3: ρ = −0.231, *P* = 0.008). However, these relationships disappeared when analyzed within each group. N2 and P3 latencies showed no significant correlations across both inhibitory processes.

Significant cross-sensory modality correlations were observed for N2 and P3 amplitudes (proactive-slowing N2, Fig.  6C: visual-auditory, ρ = 0.527, *P* < 0.001; visual-somatosensory, ρ = 0.614, *P* < 0.001; auditory-somatosensory, ρ = 0.683, *P* < 0.001; proactive-slowing P3, Fig.  6D: visual-auditory, ρ = 0.594, *P* < 0.001; visual-somatosensory, ρ = 0.580, *P* < 0.001; auditory-somatosensory, ρ = 0.712, *P* < 0.001). Cross-sensory modality correlations for proactive-slowing N2 were observed only in the ATH group (Fig.  6C: visual-auditory, ρ = 0.637, *P* = 0.001; visual-somatosensory, ρ = 0.671, *P* < 0.001; auditory-somatosensory, ρ = 0.803, *P* < 0.001), and a visual-somatosensory correlation was found in the CON group (ρ = 0.517, *P* = 0.048). Conversely, proactive-slowing P3 amplitudes showed positive correlations among sensory modalities in the CON group (Fig.  6D: visual-auditory, ρ = 0.518, *P* = 0.048; visual-somatosensory, ρ = 0.781, *P* < 0.001; auditory-somatosensory, ρ = 0.510, *P* = 0.054), and only between auditory and somatosensory modalities in the ATH group (ρ = 0.527, *P* = 0.030). Additionally, proactive-slowing P3 latency showed significant correlations across sensory modalities (visual-auditory, ρ = 0.370, *P* < 0.04; visual-somatosensory, ρ = 0.715, *P* < 0.001; auditory-somatosensory, ρ = 0.364, *P* = 0.046), whereas proactive-slowing N2 latency showed no significant correlations. These cross-sensory modality relationships of proactive-slowing P3 were observed in the CON group (visual-auditory, ρ = 0.518, *P* = 0.016; visual-somatosensory, ρ = 0.781, *P* < 0.001; auditory-somatosensory, ρ = 0.510, *P* = 0.018), and partially in the ATH group (ρ = 0.766, *P* < 0.001).

Reactive-inhibition N2 amplitude in the auditory modality was significantly correlated with both the visual (ρ = 0.384, *P* = 0.030) and somatosensory (ρ = 0.385, *P* = 0.030) modalities across all participants, but these relationships disappeared when analyzed within each group. Additionally, reactive-inhibition P3 amplitudes across sensory modalities were significantly correlated with each other (visual-auditory, ρ = 0.450, *P* = 0.006; visual-somatosensory, ρ = 0.422, *P* = 0.013; auditory-somatosensory, ρ = 0.427, *P* = 0.011), and similar correlations were observed in the visual modality with the auditory (ρ = 0.580, *P* = 0.011) and somatosensory modalities (ρ = 0.539, *P* = 0.024) in the ATH group.

## Discussion

This cross-sectional study aimed to clarify the adaptation of inhibitory control and its underlying neural mechanisms by testing two specific hypotheses: whether both proactive slowing and reactive inhibition are adaptable in shared and distinct manners, and whether supra-modal behavioral and neural adaptation is involved. These results suggest that neural adaptation associated with proactive slowing tends to be supra-modal, as evidenced by enhanced proactive slowing N2 and reduced proactive slowing P3 amplitudes in the ATH group (Fig.  5A and B). For reactive inhibition, the pattern of neural adaptation appears to be more complex. Although reactive-inhibition N2 amplitudes were significantly higher in the ATH group across all sensory modalities (Fig.  5C), suggesting supra-modal adaptation in neural activity related to conflict monitoring, no significant differences were found in reactive inhibition P3 amplitude (Fig.  5D). This may indicate that, while some neural mechanisms underlying reactive inhibition exhibit supra-modal adaptation, others may remain modality-dependent or less responsive to long-term training. Furthermore, there were no significant differences between the ATH and CON groups in the CR-T for each sensory modality, indicating that the observed adaptations of these inhibitory processes were not attributable to adaptations in sensory-motor function.

### Adaptations of proactive slowing and reactive inhibition across sensory modalities

The ATH group exhibited significantly higher proactive slowing and reactive inhibition than the CON group. While these findings suggest potential adaptations in both inhibitory processes associated with long-term, specific training, we interpret them as group differences that may reflect distinct mechanisms of inhibitory control. This cross-sectional comparison may thus offer valuable insight into how such mechanisms are shaped in trained individuals. Previously, it was unclear whether such adaptations were limited to reactive inhibition, despite evidence suggesting bidirectional behavioral adaptation specifically in reactive inhibition (Janssen et al. 2020; Albaladejo-García et al. 2023; Chan et al. 2023) and strong interactions between proactive slowing and reactive inhibition (Bissett and Logan 2014; Messel et al. 2019). To our knowledge, this is the first study to demonstrate that both proactive slowing and reactive inhibition exhibit behavioral and neural adaptations across multiple sensory modalities in athletes, likely reflecting long-term training effects.

Although inhibitory control involves both proactive slowing and reactive inhibition, these processes are supported by partially distinct neural systems. Proactive slowing reflects strategic response slowing in anticipation of stop signals, whereas reactive inhibition involves the rapid detection and resolution of conflict following stimulus onset (Braver 2012; Kenemans 2015). These processes often interact but can also dissociate under certain task demands (Aron 2011). Consistent with this dissociation, our results showed negative correlations between proactive slowing and reactive inhibition across all sensory modalities. This pattern suggests a trade-off between the two processes at the population level. Prior studies have similarly shown that Go-RT and SSRT are supported by distinct mechanisms and can vary independently under certain conditions, such as variations in stop-signal probability (Lansbergen et al. 2007), pharmacological modulation (Overtoom et al. 2009), or diagnostic group (Bekker et al. 2005). Additionally, previous research has demonstrated that proactive slowing tends to diminish when reactive inhibition is engaged (Greenhouse et al. 2012), consistent with the view that the two inhibitory processes rely on distinct mechanisms for their implementation but may be dynamically configured, such that greater reliance on one reduces the necessity for the other. These converging results reinforce the notion that proactive slowing and reactive inhibition reflect distinct neural mechanisms, each associated with differing patterns of behavioral adaptation. However, when the relationship between proactive slowing and reactive inhibition was analyzed separately within each group, the significant negative correlations observed across all participants were no longer evident. This disappearance of the relationship within each group may reflect increased variability in inhibitory control strategies among individuals. It is also possible that the across-group correlations were partly driven by pre-existing group differences, rather than reflecting consistent individual-level associations. In contrast, the presence of this relationship at the combined-group level suggests a general trade-off mechanism between proactive slowing and reactive inhibition across the population. Therefore, these findings should be interpreted cautiously, taking into account both aggregated trends and the potential influence of group-level differences, as well as within-group heterogeneity, when examining interactions between distinct inhibitory control processes.

Greater proactive slowing and reactive inhibition were observed in the ATH group across all sensory modalities, indicating that long-term, specific training results in adaptations in inhibitory control, regardless of the sensory modality. Adaptations in inhibitory control driven by visual modalities have been reported previously (Albaladejo-García et al. 2023). Moreover, studies involving athletes have shown adaptations in both proactive and reactive inhibition in response to visual stimuli (Gu et al. 2019). Athletes must process large amounts of external information based on the visual modality and make appropriate decisions rapidly, while operating in a rapidly changing environment during daily specific training. However, these studies did not clarify whether the adaptation of inhibitory control occurs in a visual modality-dependent or a cross-modal manner. So far, no consensus has been obtained regarding inhibitory control across sensory modalities (Ramautar et al. 2006; Carrillo-de-la-Peña et al. 2019; Ikarashi et al. 2022a). Therefore, the present results, which show strong correlations across sensory modalities, may indicate that inhibitory control adaptation occurs in a cross-modal manner. Furthermore, correlation analyses revealed that cross-sensory associations in proactive slowing were present not only across all participants but also within both groups, potentially suggesting a generalized mechanism underlying strategic proactive inhibitory control. Conversely, cross-sensory correlations in reactive inhibition were exclusively observed in the ATH group. This pattern suggests that proactive inhibitory control may operate across different sensory modalities, regardless of experience. In contrast, the generalization of reactive inhibition across sensory modalities may occur only with extensive training. Due to the cross-sectional nature of the present study, it was not possible to ascertain the direction of causality. However, the results suggest that long-term training may be associated with a more extensive and transferable form of inhibitory control, particularly for reactive processes.

### Neural representation for the adaptation of proactive slowing and reactive inhibition

The neurophysiological results suggest that the adaptations of proactive slowing and reactive inhibition occur through both shared and distinct neural mechanisms. The neurophysiological findings related to proactive slowing revealed significant between-group differences in proactive-slowing N2 and proactive-slowing P3 amplitudes, with greater proactive-slowing N2 amplitude and lower proactive-slowing P3 amplitude observed in the ATH group compared to the CON group. In addition, correlation analysis showed that proactive-slowing N2 and P3 amplitudes were significantly associated with proactive slowing across each sensory modality. N2 indicates an inhibitory process involved in cognitive control (Dimoska et al. 2006; Enriquez-Geppert et al. 2010). Previous studies have shown that the latency and amplitude of N2 reflect processing speed and the neural resources required to mediate executive cognitive control functions, such as response conflict and error monitoring (Smith 2011). Longer N2 latencies (Guo et al. 2018; Ikarashi et al. 2022a) and larger N2 amplitudes (Nieuwenhuis et al. 2004) have been associated with increased proactive slowing or inhibition. P3 has also been reported to reflect response inhibition and the attentional demands of a task (Kok et al. 2004). A previous study showed that a larger proactive slowing P3 amplitude indicated increased attention to response execution following the Go signal, resulting in reduced stimulus-driven proactive slowing in the visual, auditory, and somatosensory modalities (Ikarashi et al. 2022a). Therefore, it can be inferred that the adaptation of proactive slowing in this study resulted from increased neural resources allocated to conflict monitoring and decreased resources allocated to response execution.

Conversely, for the neurophysiological indices related to reactive inhibition, a significant group difference was observed only in the reactive-inhibition N2 amplitude, with a larger reactive-inhibition N2 amplitude in the ATH group compared to the CON group. Similar to the proactive slowing N2, the reactive inhibition N2 has been reported to reflect conflict evaluation and error monitoring (Ramautar et al. 2006), and/or attentional orientation to unpredicted sensory input (González-Villar et al. 2022). Therefore, increased neural resources allocated to these functions may underlie superior response inhibition in athletes. The present results differ from previous findings, suggesting that adaptations in reactive inhibition are characterized by decreased neural and attentional resource demands, as explained by the neural efficiency theory, which posits that individuals with greater cognitive or motor expertise require less neural activation to achieve the same or better performance (Chen et al. 2019; Li and Smith 2021). This may explain why the previous study reported no behavioral difference in reactive inhibition between athletes and non-athletes. In this study, superior reactive inhibition may be associated with greater reactive-inhibition N2 amplitude, although further investigation is warranted to confirm this interpretation.

Given the findings that proactive-slowing and reactive-inhibition N2 amplitudes were significantly greater in the ATH group across both inhibitory processes, and that proactive-slowing N2, reactive-inhibition N2, and proactive-slowing P3 amplitudes at frontal sites were significantly correlated, a shared neural mechanism in the frontal lobe may support adaptations in both proactive slowing and reactive inhibition. This interpretation is consistent with previous reports implicating the right inferior frontal gyrus and midfrontal regions in both inhibitory processes (van Belle et al. 2014; Gavazzi et al. 2021). Regarding the P3 component, there is an ongoing debate about its functional significance. While the P3 has traditionally been considered to reflect response inhibition and attentional demands associated with task processing (Kok et al. 2004), recent perspectives suggest that it may not directly reflect inhibitory control per se but rather the efficacy of early sensory processing (Huster et al. 2020). Furthermore, recent studies have shown that the amplitude of the stop-P3 (similar to reactive inhibition P3 in the present study) correlates with SSRT when stop-signals are highly salient, such as auditory or high-salience visual signals, suggesting that salience-enhanced inhibitory control is associated with generic rather than sensory modality-specific mechanisms (Kenemans et al. 2023; Kenemans 2025). This pattern is consistent with the theoretical framework proposed by Kenemans et al. (2023) and expanded in Kenemans (2025), which explains modality differences in stopping performance through signal salience, transmission speed, and attentional resource allocation. According to this theory, auditory stop signals—being more salient and rapidly processed—enable more efficient reactive inhibition than visual ones, which require greater top-down engagement. Our findings, particularly the modality-independent effects observed in reactive inhibition P3, may reflect an integration of modality-specific and supra-modal mechanisms supporting adaptive inhibitory control, as posited by Kenemans et al. (2023).

However, despite this theoretical alignment, caution is warranted when interpreting the reactive inhibition P3 as a definitive marker of adaptation. While the reactive-inhibition P3 may be related to reactive inhibition irrespective of sensory modality, it may not directly index the process of adaptation itself. This is because the P3 is thought to occur after the action or inhibition has been completed (Jana et al. 2020), and it is also implicated in various cognitive processes, such as attentional orienting and response conflict. Additionally, a previous study reported a clear correlation between stop-P3 amplitude and SSRT (Kenemans et al. 2023); however, the present study did not replicate this relationship. One possible explanation for this discrepancy lies in participant characteristics. Whereas Kenemans et al. (2023) primarily examined non-athlete general populations, the present study focused on kendo athletes, who may exhibit distinct levels of proactive slowing and reactive inhibition, as well as unique neural activation patterns due to long-term training. Another important consideration is the specific experimental design. Although Kenemans et al. (2023) employed only a visual Go-signal and either a visual or auditory stop-signal, the present study used visual, auditory, and somatosensory stimuli to examine both supra-modal and modality-specific effects. These differences in sensory processing demands and salience may have modulated the relationship between reactive-inhibition P3 amplitude and SSRT. Taken together, the present findings alone are insufficient to determine how P3 contributes to the adaptation of inhibitory processes. Its functional ambiguity poses a challenge in distinguishing supra-modal from modality-specific adaptations based solely on amplitude measures. Further studies combining neural and functional measures are necessary to clarify this relationship.

Moreover, the interpretation of “shared” and “distinct” neural adaptations based solely on ERP amplitudes (e.g. N2 and P3) may lack mechanistic specificity and remain speculative without further evidence. In the present study, the term “shared” is used to denote that specific ERP components (notably N2 and P3) did not demonstrate significant variations across sensory modalities or inhibitory processes, which may imply a supra-modal adaptation. However, such interpretations should be approached with caution, as they rely on surface-level comparisons rather than direct evidence of common neural sources. Future research employing source localization, multivariate pattern analysis, or connectivity-based approaches will be essential to determine whether these components indeed reflect overlapping neural mechanisms. Furthermore, variations in ERP waveforms and amplitudes across sensory modalities and task conditions suggest that different neural networks may be engaged, or that a common network may be flexibly recruited in a context-dependent manner (Tanaka et al. 2008; Huster et al. 2010; Swick et al. 2011). While the present findings provide initial evidence for both modality-specific and supra-modal patterns in N2 and P3 responses, further research is required to determine whether these patterns reflect the engagement of distinct neural circuits or the dynamic reconfiguration of a shared inhibitory control network.

Although no significant correlations were found between the ERP components (N2 and P3) and behavioral indices of inhibitory control, these exploratory analyses aimed to determine whether neurophysiological markers relate to individual differences in performance. The absence of significant associations may reflect the limited sensitivity of ERP amplitudes in capturing the complex dynamics of inhibitory control, or it may suggest that proactive and reactive inhibition engage distinct neural mechanisms, making simple linear relationships unlikely. Future studies employing more advanced analytic techniques—such as trial-by-trial modeling, dynamic causal modeling, or connectivity analyses—may better elucidate the neural basis of individual variability in inhibitory function.

## Implications and limitations

This study offers novel insights into the adaptation of proactive slowing and reactive inhibition across sensory modalities in humans. These findings have methodological implications for neurophysiological inquiries into human cognitive function, wherein the adaptation of proactive slowing and reactive inhibition should be analyzed separately when examining adaptations of IC, as both shared and distinct neural mechanisms are involved in the two inhibitory modes. Additionally, the present findings suggest that the adaptation of both proactive slowing and reactive inhibition involves not only shared but also distinct neural mechanisms. Therefore, the choice of sensory modality when examining the adaptation of inhibitory control may critically influence methodological decisions and the interpretation of the underlying neural mechanisms. In this study, due to their extensive training-induced functional adaptations, sports athletes—especially those engaged in competitive interpersonal sports such as kendo—were studied as a unique population in research investigating behavioral adaptation and neural representation. As expected, the ATH group exhibited greater proactive slowing and reactive inhibition. Given that IC has been reported to decline with age and disease, the present results suggest that regular daily exercise may play a role in improving or maintaining overall inhibitory function. Furthermore, the present findings have potential practical implications for sports training and athlete development. Understanding that both proactive and reactive inhibition can adapt across sensory modalities suggests that cognitive training programs targeting IC could be designed to enhance athletic performance. For example, training athletes to flexibly switch between proactive and reactive strategies depending on situational demands may improve decision-making speed and accuracy in dynamic sport environments. Moreover, these insights may assist in developing rehabilitation strategies for athletes recovering from injuries by focusing on restoring or strengthening specific aspects of cognitive control that are crucial for a safe and effective return to play.

However, several issues remain to be addressed before such conclusions can be drawn. First, this study recruited Kendo athletes to examine differences in inhibitory control adaptation. However, prior research has indicated that athletes in open-skill interpersonal competitions demonstrate superior inhibitory control (Taddei et al. 2012; Zhang et al. 2015; Brevers et al. 2018). It is important to note that Kendo athletes are specifically trained to initiate actions reactively in response to an opponent’s movements, rather than proactively initiating them. This sport-specific characteristic may have influenced the longer Go reaction times (Go-RTs) observed in the SST. Importantly, longer Go-RTs may result from either proactive slowing, which reflects an internally regulated inhibitory modulation of response execution, or a waiting strategy, in which participants consciously withhold their responses until they are confident that no stop signal will occur. The former is regarded as a behavioral manifestation of proactive inhibitory control, whereas the latter represents a conscious strategic adjustment that studies typically attempt to minimize through explicit instructions and tracking procedures. Although we instructed participants not to wait and employed a tracking procedure to discourage this behavior, we cannot fully exclude the possibility that sport-specific tendencies or strategic behaviors influenced the results. Moreover, because only Kendo athletes were included, it remains unclear whether these findings can be generalized to all contexts of inhibitory function adaptation. Future studies should include a diverse range of individuals with response inhibition adaptations—such as athletes from other sports, musicians, or patients with mental disorders—to validate and expand these findings. Second, this study was a cross-sectional investigation involving participants whose adaptation had already occurred. Although we used the term “adaptation” to describe the observed group differences, we acknowledge that the cross-sectional nature of the study precludes causal inference regarding training-induced changes. The present findings should therefore be interpreted as evidence of group differences in inhibitory control potentially associated with long-term training, rather than as direct evidence of neural plasticity. Future longitudinal or interventional studies are needed to confirm whether these differences result from training or pre-existing traits. Third, longer Go-RTs may reflect a waiting strategy, whereby participants deliberately delay their responses until they are confident that no stop signal will appear. Although this behavior superficially resembles enhanced proactive slowing, the two are conceptually and mechanistically distinct: proactive slowing reflects internally regulated inhibition, whereas waiting is a strategic response based on task prediction. We attempted to minimize the influence of waiting strategies through participant instructions and an adaptive tracking procedure; however, their complete exclusion cannot be guaranteed. Notably, such strategic delay is unlikely to affect reactive inhibition measures because the SSD varied dynamically and the probability of stop trials was maintained at approximately 50% (Rieger and Gauggel 2002). Fourth, the stimulus durations differed across the visual, auditory, and somatosensory modalities. These durations were selected based on prior studies evaluating ERPs within each modality (Ramautar et al. 2006; Carrillo-de-la-Peña et al. 2019; Ikarashi et al. 2022a) and were adjusted to ensure clear perception and to avoid discomfort, particularly for the somatosensory stimuli. However, differences in stimulus saliency and temporal characteristics may have influenced sensory processing and response inhibition measures, such as SSRT. Future studies should consider standardizing or systematically manipulating stimulus durations across modalities to better isolate modality-specific effects on inhibitory control. Fifth, the present study focused exclusively on ERP components as time-locked neural markers of proactive and reactive inhibition. While mid-frontal theta and posterior alpha oscillations are well-established markers of conflict monitoring and attentional modulation, respectively, we did not perform time–frequency analyses due to methodological constraints. Specifically, the use of a 10-channel EEG system precluded source-level analyses and reliable topographical localization of frequency-specific activity, such as ICA or dipole modeling, which are essential for interpreting oscillatory dynamics. Thus, although these frequency-domain markers may provide additional insights into the neural mechanisms of inhibitory control, we decided not to include them in the present study. Future investigations employing high-density EEG and dedicated time–frequency methods are required to explore these aspects more comprehensively. Sixth, although early sensory processing components such as P1 and N1 may also be relevant to inhibitory control, the present study did not include these ERP measures, which are typically associated with early-stage perceptual processing. Nonetheless, understanding the contribution of early sensory processing to the adaptation of inhibitory control remains an important open question. Future studies employing high-density EEG or multimodal neuroimaging techniques are warranted to clarify the temporal dynamics underlying proactive and reactive inhibition. Seventh, another limitation concerns the potential contamination of stop-signal-induced ERPs by overlapping Go-related activity. Although the present study employed SS–US difference waves to minimize such overlap, residual Go-ERP contributions—particularly in the baseline interval (−100 to 0 ms)—may have influenced the resulting ERP waveforms. This issue has been highlighted in prior work (Bekker et al. 2005), which proposed advanced correction techniques such as ADJAR-level 2 filtering to address this problem more thoroughly. While the current findings remain interpretable, future studies should consider implementing such methods to more precisely isolate reactive-inhibition ERP components and enhance the mechanistic interpretation of reactive inhibition. Finally, due to the experimental design of the present study, in which the SST was divided into three separate blocks, it was difficult to estimate trigger failures reliably using standard methods (Heathcote et al. 2019; Skippen et al. 2020). Future studies employing continuous SST designs are warranted to better assess the role of trigger failures in proactive and reactive inhibitory control.

## Conclusion

This cross-sectional study provides novel evidence that both proactive slowing and reactive inhibition exhibit behavioral adaptations across sensory modalities, accompanied by neural adaptations that are partially shared and partially distinct.

## References

[ref1] Alatorre-Cruz GC et al. 2021. Effect of obesity on inhibitory control in preadolescents during stop-signal task. An event-related potentials study. Int J Psychophysiol. 165:56–67. 10.1016/j.ijpsycho.2021.04.003. 33872629

[ref2] Albaladejo-García C, García-Aguilar F, Moreno FJ. 2023. The role of inhibitory control in sport performance: systematic review and meta-analysis in stop-signal paradigm. Neurosci Biobehav Rev. 147:105108. 10.1016/j.neubiorev.2023.105108. 36828162

[ref3] Aron AR . 2011. From reactive to proactive and selective control: developing a richer model for stopping inappropriate responses. Biol Psychiatry. 69:e55–e68. 10.1016/j.biopsych.2010.07.024. 20932513 PMC3039712

[ref4] Bari A, Robbins TW. 2013. Inhibition and impulsivity: behavioral and neural basis of response control. Prog Neurobiol. 108:44–79. 10.1016/j.pneurobio.2013.06.005. 23856628

[ref5] Bekker EM, Kenemans JL, Hoeksma MR, Talsma D, Verbaten MN. 2005. The pure electrophysiology of stopping. Int J Psychophysiol. 55:191–198. 10.1016/j.ijpsycho.2004.07.005. 15649550

[ref7] Bissett PG, Logan GD. 2014. Selective stopping? Maybe not. J Exp Psychol Gen. 143:455–472. 10.1037/a0032122. 23477668 PMC3728275

[ref8] Braver TS . 2012. The variable nature of cognitive control: a dual mechanisms framework. Trends Cogn Sci. 16:106–113. 10.1016/j.tics.2011.12.010. 22245618 PMC3289517

[ref9] Bravi R et al. 2022. Effect of different sport environments on proactive and reactive motor inhibition: a study on open- and closed-skilled athletes via mouse-tracking procedure. Front Psychol. 13:1042705. 10.3389/fpsyg.2022.1042705. 36578693 PMC9791124

[ref10] Brevers D et al. 2018. Proactive and reactive motor inhibition in top athletes versus nonathletes. Percept Mot Skills. 125:289–312. 10.1177/0031512517751751. 29310525

[ref11] Carrillo-de-la-Peña MT, Bonilla FM, González-Villar AJ. 2019. Effect of the stop-signal modality on brain electrical activity associated with suppression of ongoing actions. Biol Psychol. 143:85–92. 10.1016/j.biopsycho.2019.01.010. 30807785

[ref12] Chan CC et al. 2023. Neural correlates of impulsivity in bipolar disorder: a systematic review and clinical implications. Neurosci Biobehav Rev. 147:105109. 10.1016/j.neubiorev.2023.105109. 36813146 PMC11073484

[ref13] Chen J et al. 2019. Enhanced inhibitory control during re-engagement processing in badminton athletes: an event-related potential study. J Sport Health Sci. 8:585–594. 10.1016/j.jshs.2019.05.005. 31720072 PMC6834996

[ref14] Chikazoe J et al. 2009. Preparation to inhibit a response complements response inhibition during performance of a stop-signal task. J Neurosci. 29:15870–15877. 10.1523/JNEUROSCI.3645-09.2009. 20016103 PMC6666181

[ref15] Cohen J. 2013. Statistical power analysis for the behavioral sciences. 2nd ed. London, England: Routledge. 10.4324/9780203771587.

[ref16] Colzato LS, Hertsig G, van den Wildenberg WPM, Hommel B. 2010. Estrogen modulates inhibitory control in healthy human females: evidence from the stop-signal paradigm. Neuroscience. 167:709–715. 10.1016/j.neuroscience.2010.02.029. 20219635

[ref17] Corbetta M, Patel G, Shulman GL. 2008. The reorienting system of the human brain: from environment to theory of mind. Neuron. 58:306–324. 10.1016/j.neuron.2008.04.017. 18466742 PMC2441869

[ref18] Devor T et al. 2025. Reduced theta inter-trial phase coherence in error processing: a marker of neural dysfunction in attention deficit hyperactivity disorder. Psychophysiology. 62:e14764. 10.1111/psyp.14764. 39817345 PMC11736539

[ref19] Diamond A . 2013. Executive functions. Annu Rev Psychol. 64:135–168. 10.1146/annurev-psych-113011-143750. 23020641 PMC4084861

[ref20] Dimoska A, Johnstone SJ, Barry RJ. 2006. The auditory-evoked N2 and P3 components in the stop-signal task: indices of inhibition, response-conflict or error-detection? Brain Cogn. 62:98–112. 10.1016/j.bandc.2006.03.011. 16814442

[ref21] Enriquez-Geppert S, Konrad C, Pantev C, Huster RJ. 2010. Conflict and inhibition differentially affect the N200/P300 complex in a combined go/nogo and stop-signal task. NeuroImage. 51:877–887. 10.1016/j.neuroimage.2010.02.043. 20188191

[ref22] Falkenstein M, Hoormann J, Hohnsbein J. 2002. Inhibition-related ERP components: variation with modality, age, and time-on-task. J Psychophysiol. 16:167–175. 10.1027/0269-8803.16.3.167.

[ref23] Fleddermann M-T, Reichert L, Wieland B, Zentgraf K. 2023. Stop it! Relationship between sport expertise and response inhibition in elite athletes. Front Psychol. 14:1192483. 10.3389/fpsyg.2023.1192483. 37342635 PMC10278942

[ref24] Gavazzi G, Giovannelli F, Currò T, Mascalchi M, Viggiano MP. 2021. Contiguity of proactive and reactive inhibitory brain areas: a cognitive model based on ALE meta-analyses. Brain Imaging Behav. 15:2199–2214. 10.1007/s11682-020-00369-5. 32748318 PMC8413163

[ref25] González-Villar A, Galdo-Álvarez S, Carrillo-de-la-Peña MT. 2022. Neural correlates of unpredictable stop and non-stop cues in overt and imagined execution. Psychophysiology. 59:e14019. 10.1111/psyp.14019. 35224733 PMC9286458

[ref26] Greenhouse I, Oldenkamp CL, Aron AR. 2012. Stopping a response has global or nonglobal effects on the motor system depending on preparation. J Neurophysiol. 107:384–392. 10.1152/jn.00704.2011. 22013239 PMC3349702

[ref27] Gu Q, Zou L, Loprinzi PD, Quan M, Huang T. 2019. Effects of open versus closed skill exercise on cognitive function: a systematic review. Front Psychol. 10:1707. 10.3389/fpsyg.2019.01707. 31507472 PMC6718477

[ref27a] Guo Z et al. 2018. The impairing effects of mental fatigue on response inhibition: An ERP study. PLoS One. 13:e0198206. 10.1371/journal.pone.0198206. PMC5983454 29856827

[ref28] Heathcote A et al. 2019. Dynamic models of choice. Behav Res Methods. 51:961–985. 10.3758/s13428-018-1067-y. 29959755

[ref29] Heppe H, Zentgraf K. 2019. Team handball experts outperform recreational athletes in hand and foot response inhibition: a Behavioral study. Front Psychol. 10:971. 10.3389/fpsyg.2019.00971. 31133925 PMC6524689

[ref30] Hester RL et al. 2004. Predicting success: patterns of cortical activation and deactivation prior to response inhibition. J Cogn Neurosci. 16:776–785. 10.1162/089892904970726. 15200705

[ref31] Huster RJ, Westerhausen R, Pantev C, Konrad C. 2010. The role of the cingulate cortex as neural generator of the N200 and P300 in a tactile response inhibition task. Hum Brain Mapp. 31:1260–1271. 10.1002/hbm.20933. 20063362 PMC6871040

[ref32] Huster RJ, Messel MS, Thunberg C, Raud L. 2020. The P300 as marker of inhibitory control - fact or fiction? Cortex. 132:334–348. 10.1016/j.cortex.2020.05.021. 33017748

[ref33] Ikarashi K, Sato D, Fujimoto T, Edama M, Baba Y, Yamashiro K. 2022a. Response inhibitory control varies with different sensory modalities. Cereb Cortex. 32:275–285. 10.1093/cercor/bhab207. 34223874

[ref34] Ikarashi K, Sato D, Ochi G, Fujimoto T, Yamashiro K. 2022b. Action postponing and restraint varies among sensory modalities. Brain Sci. 12:1530. 10.3390/brainsci12111530. PMC9688532 36421854

[ref35] Jana S, Hannah R, Muralidharan V, Aron AR. 2020. Temporal cascade of frontal, motor and muscle processes underlying human action-stopping. elife. 9:e50371. 10.7554/eLife.50371. PMC7159878 32186515

[ref36] Janssen TWP, van Atteveldt N, Oosterlaan J. 2020. Error and post-error processing in children with attention-deficit/hyperactivity disorder: an electrical neuroimaging study. Clin Neurophysiol. 131:2236–2249. 10.1016/j.clinph.2020.06.022. 32721844

[ref37] Kenemans JL . 2015. Specific proactive and generic reactive inhibition. Neurosci Biobehav Rev. 56:115–126. 10.1016/j.neubiorev.2015.06.011. 26116545

[ref38] Kenemans JL . 2025. Action cancellation and salience of the cancellation signal. JoCN Forum. 10.21428/8e6ba8ef.70474aa5.

[ref39] Kenemans JL, Schutte I, Van Bijnen S, Logemann HNA. 2023. How salience enhances inhibitory control: an analysis of electro-cortical mechanisms. Biol Psychol. 177:108505. 10.1016/j.biopsycho.2023.108505. 36669616

[ref40] Kok A, Ramautar JR, De Ruiter MB, Band GPH, Ridderinkhof KR. 2004. ERP components associated with successful and unsuccessful stopping in a stop-signal task. Psychophysiology. 41:9–20. 14692996 10.1046/j.1469-8986.2003.00127.x

[ref41] Lakens D . 2022. Sample size justification. Collabra Psychol. 8:33267. 10.1525/collabra.33267.

[ref42] Lakens D, Caldwell AR. 2021. Simulation-based power analysis for factorial analysis of variance designs. Adv Methods Pract Psychol Sci. 4:2515245920951503. 10.1177/2515245920951503.

[ref43] Lansbergen MM, Böcker KBE, Bekker EM, Kenemans JL. 2007. Neural correlates of stopping and self-reported impulsivity. Clin Neurophysiol. 118:2089–2103. 10.1016/j.clinph.2007.06.011. 17652017

[ref44] Li L, Smith DM. 2021. Neural efficiency in athletes: a systematic review. Front Behav Neurosci. 15:698555. 10.3389/fnbeh.2021.698555. 34421553 PMC8374331

[ref45] Logan GD, Cowan WB, Davis KA. 1984. On the ability to inhibit simple and choice reaction time responses: a model and a method. J Exp Psychol Hum Percept Perform. 10:276–291. 10.1037/0096-1523.10.2.276. 6232345

[ref46] Luck SJ . 2014. An introduction to the event-related potential technique. In: A Bradford book. 2nd ed. Cambridge, MA: Bradford Books.

[ref47] Luck SJ, Gaspelin N. 2017. How to get statistically significant effects in any ERP experiment (and why you shouldn’t). Psychophysiology. 54:146–157. 10.1111/psyp.12639. 28000253 PMC5178877

[ref48] McKay AKA et al. 2022. Defining training and performance Caliber: a participant classification framework. Int J Sports Physiol Perform. 17:317–331. 10.1123/ijspp.2021-0451. 34965513

[ref49] Messel MS, Raud L, Hoff PK, Skaftnes CS, Huster RJ. 2019. Strategy switches in proactive inhibitory control and their association with task-general and stopping-specific networks. Neuropsychologia. 135:107220. 10.1016/j.neuropsychologia.2019.107220. 31586553

[ref50] Moreno S et al. 2011. Short-term music training enhances verbal intelligence and executive function. Psychol Sci. 22:1425–1433. 10.1177/0956797611416999. 21969312 PMC3449320

[ref51] Nieuwenhuis S, Yeung N, Cohen JD. 2004. Stimulus modality, perceptual overlap, and the go/no-go N2. Psychophysiology. 41:157–160. 10.1046/j.1469-8986.2003.00128.x. 14693011

[ref52] Overtoom CCE et al. 2009. Methylphenidate restores link between stop-signal sensory impact and successful stopping in adults with attention-deficit/hyperactivity disorder. Biol Psychiatry. 65:614–619. 10.1016/j.biopsych.2008.10.048. 19103443

[ref53] Polich J . 2007. Updating P300: an integrative theory of P3a and P3b. Clin Neurophysiol. 118:2128–2148. 10.1016/j.clinph.2007.04.019. 17573239 PMC2715154

[ref54] Ramautar JR, Kok A, Ridderinkhof KR. 2006. Effects of stop-signal modality on the N2/P3 complex elicited in the stop-signal paradigm. Biol Psychol. 72:96–109. 10.1016/j.biopsycho.2005.08.001. 16157441

[ref65] RC Team . 2013. R: a language and environment for statistical computing. https://cran.r-project.org/doc/manuals/r-release/fullrefman.pdf.

[ref55] Rieger M, Gauggel S. 2002. Inhibition of ongoing responses in patients with traumatic brain injury. Neuropsychologia. 40:76–85. 10.1016/S0028-3932(01)00068-9. 11595263

[ref6] van Belle J, Vink M, Durston S, Zandbelt BB. 2014. Common and unique neural networks for proactive and reactive response inhibition revealed by independent component analysis of functional MRI data. NeuroImage. 103:65–74. 10.1016/j.neuroimage.2014.09.014. 25224995

[ref56] van Rooij D, van Bijnen S, Schutte I, van der Stoep N, Kenemans JL. 2024. Frontal theta power prospectively associated with response inhibition. bioRxiv. 10.1101/2024.05.13.593803.

[ref57] Senderecka M, Szewczyk J, Wichary S, Kossowska M. 2018. Individual differences in decisiveness: ERP correlates of response inhibition and error monitoring. Psychophysiology. 55:e13198. 10.1111/psyp.13198. 29781210

[ref58] Skippen P et al. 2020. Reconsidering electrophysiological markers of response inhibition in light of trigger failures in the stop-signal task. Psychophysiology. 57:e13619. 32725926 10.1111/psyp.13619

[ref59] Smith JL . 2011. To go or not to go, that is the question: do the N2 and P3 reflect stimulus- or response-related conflict? Int J Psychophysiol. 82:143–152. 10.1016/j.ijpsycho.2011.07.019. 21851842

[ref60] Stuphorn V, Emeric EE. 2012. Proactive and reactive control by the medial frontal cortex. Front Neuroeng. 5:9. 22723779 10.3389/fneng.2012.00009 PMC3378012

[ref61] Swick D, Ashley V, Turken U. 2011. Are the neural correlates of stopping and not going identical? Quantitative meta-analysis of two response inhibition tasks. NeuroImage. 56:1655–1665. 10.1016/j.neuroimage.2011.02.070. 21376819

[ref62] Taddei F, Bultrini A, Spinelli D, Di Russo F. 2012. Neural correlates of attentional and executive processing in middle-age fencers. Med Sci Sports Exerc. 44:1057–1066. 10.1249/MSS.0b013e31824529c2. 22157879

[ref63] Tanaka E et al. 2008. A transition from unimodal to multimodal activations in four sensory modalities in humans: an electrophysiological study. BMC Neurosci. 9:116. 19061523 10.1186/1471-2202-9-116 PMC2607283

[ref64] Tatz JR, Soh C, Wessel JR. 2021. Common and unique inhibitory control signatures of action-stopping and attentional capture suggest that actions are stopped in two stages. J Neurosci. 41:8826–8838. 10.1523/JNEUROSCI.1105-21.2021. 34493541 PMC8528501

[ref66] Verbruggen F et al. 2019. A consensus guide to capturing the ability to inhibit actions and impulsive behaviors in the stop-signal task. elife. 8:e46323. 10.7554/eLife.46323. PMC6533084 31033438

[ref67] van den Wildenberg WPM, Ridderinkhof KR, Wylie SA. 2022. Towards conceptual clarification of proactive inhibitory control: a review. Brain Sci. 12:1638. 10.3390/brainsci12121638. PMC9776056 36552098

[ref67a] Wang Y et al. 2024. Dorsolateral prefrontal cortex to ipsilateral primary motor cortex intercortical interactions during inhibitory control enhance response inhibition in open-skill athletes. Sci Rep. 2024;14:24345. 10.1038/s41598-024-75151-4. PMC11487194 39420010

[ref68] Xu L et al. 2022. Acute sleep deprivation impairs motor inhibition in table tennis athletes: an ERP study. Brain Sci. 12:746. 10.3390/brainsci12060746. 35741631 PMC9221109

[ref69] Yamashiro K et al. 2023. Transcranial high-frequency random noise stimulation does not modulate Nogo N2 and go/Nogo reaction times in somatosensory and auditory modalities. Sci Rep. 13:3014. 36810889 10.1038/s41598-023-30261-3 PMC9944265

[ref70] Zandbelt BB, Vink M. 2010. On the role of the striatum in response inhibition. PLoS One. 5:e13848. 10.1371/journal.pone.0013848. 21079814 PMC2973972

[ref71] Zhang D, Ding H, Wang X, Qi C, Luo Y. 2015. Enhanced response inhibition in experienced fencers. Sci Rep. 5:16282. 10.1038/srep16282. 26541899 PMC4635338

[ref72] Zuk J, Benjamin C, Kenyon A, Gaab N. 2014. Behavioral and neural correlates of executive functioning in musicians and non-musicians. PLoS One. 9:e99868. 10.1371/journal.pone.0099868. 24937544 PMC4061064

